# Investigating psychometric properties of the arm activity measure – Thai version (ArmA-TH) sub‐scales using the Rasch model

**DOI:** 10.1186/s12874-021-01238-5

**Published:** 2021-03-09

**Authors:** Montana Buntragulpoontawee, Jeeranan Khunachiva, Patreeya Euawongyarti, Nahathai Wongpakaran, Tinakon Wongpakaran, Atcharee Kaewma, Stephen Ashford

**Affiliations:** 1 Department of Rehabilitation Medicine, Faculty of Medicine, Chiang Mai University, 50200 Chiang Mai, Thailand; 2 Department of Psychiatry, Faculty of Medicine, Chiang Mai, 50200 Chiang Mai, Thailand; 3 Department of Rehabilitation Medicine, Nakornping Hospital, Chiang Mai, Thailand; 4 Regional Hyper-acute Rehabilitation Unit, London North West University Healthcare NHS Trust, London, UK; 5 Centre for Nursing, Midwifery and Allied Health Research, University College London Hospitals, University College London, London, UK; 6 Department of Palliative Care, Policy and Rehabilitation, King’s College London, London, UK

**Keywords:** ArmA-TH, Rasch analysis, Psychometric properties, Upper limb hemiplegia, Outcome measure

## Abstract

**Background:**

This study investigated the ArmA-TH sub-scale measurement properties based on item response theory using the Rasch model.

**Methods:**

Patients with upper limb hemiplegia resulting from cerebrovascular and other brain disorders were asked to complete the ArmA-TH questionnaire. Rasch analysis was performed to test how well the ArmA-TH passive and active function sub-scales fit the Rasch model by investigating unidimensionality, response category functioning, reliability of person and item, and differential item functioning (DIF) for age, sex, and education.

**Results:**

Participants had stroke or other acquired brain injury (n = 185), and the majority were men (126, 68.1 %), with a mean age of 55 (SD 22). Most patients (91, 49.2 %) had graduated from elementary/primary school. For the ArmA-TH passive function scale, all items had acceptable fit statistics. The scale’s unidimensionality and local independence were supported. The reliability was acceptable. A disordered threshold was found for five items, and none indicated DIF. For the ArmA-TH active function scale, one item was misfit and three were locally dependent. The reliability was good. No items showed DIF. All items had disordered thresholds, and the data fitted the Rasch model better after rescoring.

**Conclusions:**

Both sub-scales of ArmA-TH fitted the Rasch model and were valid and reliable. The disordered thresholds should be further investigated.

## Background

The Arm Activity Measure questionnaire (ArmA) is a twenty-item patient and/or carer-reported outcome measure of function of hemiparetic upper limbs developed in 2013 by Ashford et al. with the primary goal of addressing ‘real–life’ function, that is, day-to-day performance in the person’s normal environment [1]. The unique characteristic of ArmA is its two separate constructs, as it has passive and active function sub-scales, for evaluating the most clinically relevant goals [2]. As there has never been an objective self-report measure for assessing hemiparetic upper limb function for patients in Thailand, ArmA was translated into the Thai language as ArmA-TH with a preliminary evaluation of psychometric properties, including a content validity index for both item (I-CVI) and score (S-CVI), inter-rater reliability, and internal consistency [3]. However, neither the construct validity of the items nor a detailed item evaluation of ArmA-TH based on measurement theory were initially explored. According to Rasch measurement theory, an outcome measure scale should demonstrate unidimensionality (all items contribute to the same construct) and have no DIF (invariance across a sub-population) [4]. All these properties can be evaluated by conformity to the Rasch model, which the original English version of the ArmA passive function sub-scale was evaluated against in a UK sample [5].

In this study, we therefore aimed to examine the extent to which our data, from a Thai sample, fit the Rasch measurement model. The Rasch model is an item-response latent trait model, which is a probabilistic logistic model that predicts that the response to a particular item is influenced by the quality of both the person and the item. The key concepts of the Rasch model are, first, that it transforms non-linear raw scores into logit scale measures, in which the location (logit) of both the particular person and the item are determined on the same interval scale. This interval scale can differentiate how people adhere to the fundamental measurement principle, which provides interval-level measurement as opposed to ordinal scaling using the raw score [6].

The second concept is ‘local independence’ [4, 6], which implies that there should not be any correlation between two items after the effect of the latent trait is conditioned out (the correlation of residuals should be zero) [7]. Violation of local independence can affect unidimensionality, and both local independence and DIF are important to differentiating the individual as a function of latent trait scores.

## Methods

### Population

Patients with hemiplegic upper limb impairment resulting from stroke or other acquired brain injury who were receiving rehabilitation services in Chiang Mai, Thailand were asked to complete the ArmA-TH questionnaire in person. All patients were asked to give written informed consent before proceeding with the questionnaire. The patients were between 20 and 85 years of age, had Thai as their mother tongue, and had graduated from at least elementary school with the ability to understand Thai communication for daily activities. The patient demographic characteristics in this study were age, sex, hemiparetic side, diagnosis, education level, and ArmA-TH passive and active scores. Ethical approval for the research programme was received from the Research Ethics Committee of Faculty of Medicine, Chiang Mai University, Thailand, Ethic approval number REH-2558-03109.

### Measure

The ArmA-TH is a twenty-item questionnaire for assessing the difficulty in functioning of a hemiparetic upper limb. There are seven items in the passive function sub-scale and thirteen items in the active function sub-scale. Using a Likert scoring system between 0 (no difficulty) and 4 (unable to do task), the passive function sub-scale scores range from 0 (high function) to 28, and the active function sub-scale scores range from 0 (high function) to 52 [2].

### Analysis

Descriptive statistics were used to describe the demographic characteristics of the patients, presented as mean (SD). The ArmA-TH sub-scale scores are presented in terms of median (interquartile range).

### Rasch analysis

- Partial credit Rasch model was used for analysis, the following criteria were investigated [8, 9].

1. Unidimensionality. Two methods were evaluated for determining unidimensionality. First, the first principal component of the residuals (first construct) should be no more than 15 % or have an eigenvalue less than 2 [10]. Second, that the item fit statistics were assessed using outfit and infit mean-square statistics. Outfit mean-square is calculated by averaging the squared residuals for each item across all persons, whereas, infit values was computed by having squared residuals to be weighted by their variances before averaging. The outfit MNSQ and infit MNSQ should be 0.70 and 1.50 [10]. In addition, the correlation of the two sets of person measures and the correlation disattenuated due to measurement error should be greater than 0.7 to indicate unidimensionality.
2. Local independence. To evaluate local independency, a pair of items should not have inter-item residual correlations higher than 0.2 [11].
3. Reliability. There are two kinds of reliability evaluated by Rasch analysis: person reliability and item reliability. The person reliability is interpreted as the ability of the scale to reliably rank the person relative to location within the scale of the measure. Similar to Cronbach’s alpha, but the value of person reliability is often lower than that of Cronbach’s because it does not include extreme scores. The item reliability coefficient reflects the extent to which the item hierarchy is replicable with a different set of individuals. A reliability coefficient of > 0.70 is considered acceptable for a person, and a coefficient of ≥ 0.80 is considered acceptable for an item.

Response category functioning. Ordered categories and thresholds are expected for measurement. Therefore, adjacent categories (thresholds) on the latent scale have the same position and order on the latent trait measured [12]. Items with a disordered threshold between categories can be evaluated by category probability curves, and the item fit of each categorical response is examined. Item fits less than 2.0 are acceptable) [8]. Items that exhibited disordered thresholds were rescored by collapsing adjacent categories, and a reanalysis was performed to check whether it showed a better fit to the model.

(4) Targeting of persons, items, and item hierarchy. Acceptable item-test targeting for compliance with the Rasch model is evaluated through the closeness of the mean of the person and the mean of the item on the Wright map (no more than 1 logit) [13]. The item hierarchy indicates how the items match the intentions of the instrument developer in difficulty and the expectations of those planning to use the test results [14].
(5) DIF for age, sex, and education. An ideal item is one with invariant measurement properties across subgroups, meaning that item calibration should be the same in different subgroups of people [8]. Moderate-to-large DIF was evaluated using a significant DIF contrast of < 0.64, thereby indicating an acceptable value [6]. In this study, DIF due to age, sex, and education was examined. Both the ArmA-TH passive and active function sub-scales were separately evaluated for fit to the Rasch model.

Winsteps 4.7.1.0 (Winsteps® Rasch Measurement, 2017) was used for the Rasch analysis.

## Results

A total of 185 patients participated in the questionnaire evaluation. The majority were men (126, 68.1 %) with mean age of 55 (SD 22). The hemiparesis resulted from haemorrhagic stroke (81, 43.8 %), ischemic stroke (78, 42.1 %), traumatic brain injury (24, 13.0 %) and other causes (2, 1.1 %). Most patients (91, 49.2 %) had graduated at the elementary/primary school level, followed by secondary school level (40, 21.6 %), vocational or high vocational certificate (28, 15.1 %), and the smallest group had a bachelor’s degree or above (26, 14.1 %). The ArmA-TH passive function sub-scale scores ranged from 0 to 28, covering the total range from minimum to maximum score. The ArmA-TH active function sub-scale scores ranged from a minimum of 0 to 49, almost reaching the maximum score of 52. Details are shown in Table 1.

**Table 1:** Demographic characteristics and ArmA-TH sub-scale scores of 185 patients completing the ArmA-TH questionnaire

**Demographic characteristics**	**Number (%)*(n=185)***	
**Mean age (years)**	55	(SD 22.0)
**Sex **		
Male	126	(68.1)
Female	59	(31.9)
**Diagnosis**		
Haemorrhagic stroke	81	(43.8)
Ischemic stroke	78	(42.1)
Traumatic brain injury	24	(13.0)
Other brain injury	2	(1.1)
**Education**		
Primary school	91	(49.2)
Secondary school	40	(21.6)
Vocational or high vocational certificate	28	(15.1)
Bachelor’s degree and above	26	(14.1)
**ArmA**	Median (Interquartile range)	
Passive Function Sub-Scale	6	(2-11)
Active Function Sub-Scale	11	(5-18)

### Analysis according to the Rasch model

#### ArmA-TH passive function

For the ArmA-TH passive function, the fit statistics ranged from 0.73 to 1.31, indicating all items contributed to the Rasch measurement model. Principal component analysis of the residuals showed the first eigenvalue of 1.73 (11.5 %) supported unidimensionality, whereas the standardized residual correlations were less than 0.3, indicating local independence.

**Fig. 1:**
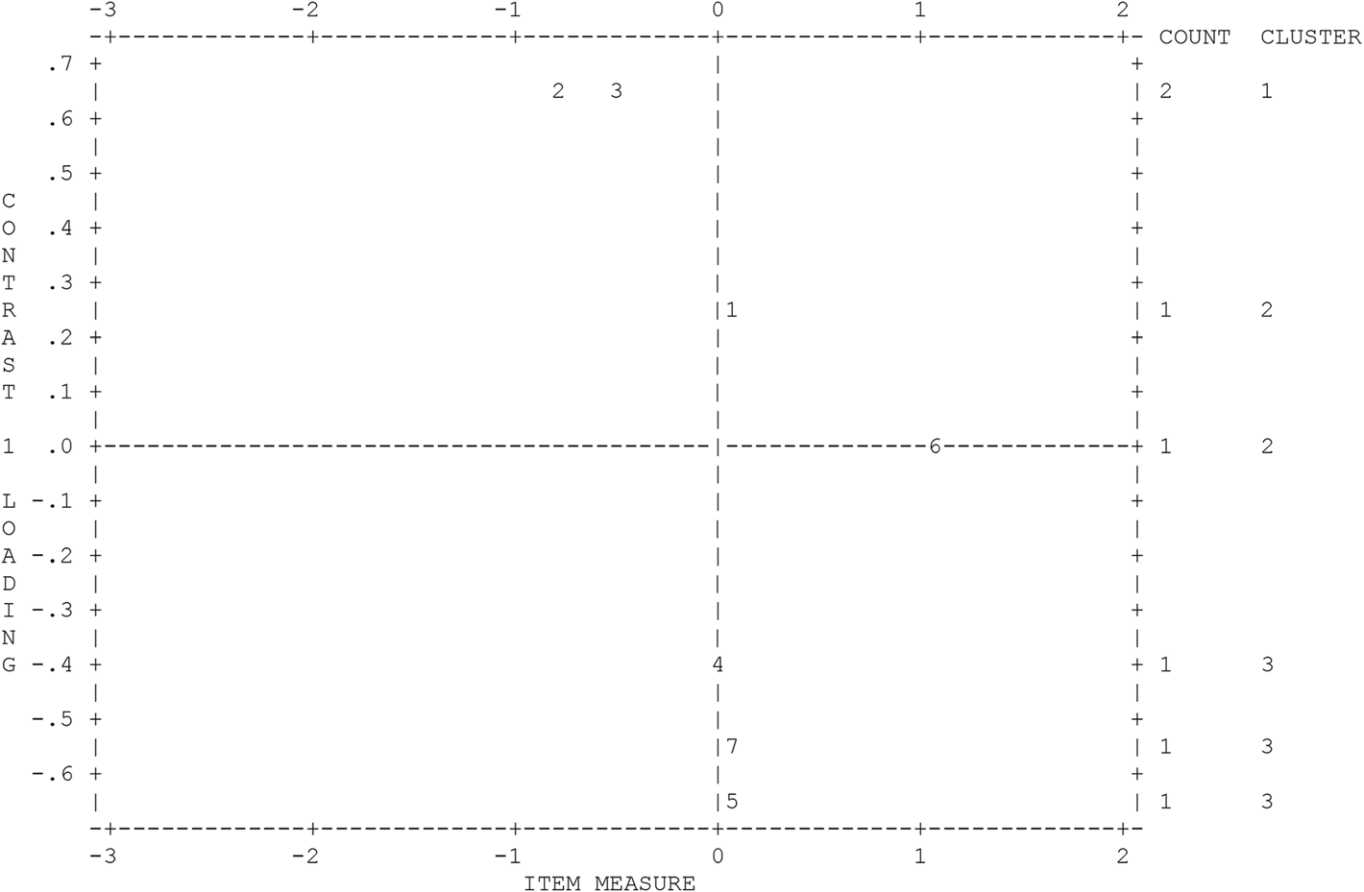
Plots of item loadings on the first factor of the passive function scale. Legend: Numbers 1–7 represent items 1–7 of the passive function scale. Figure created by Winsteps 4.7.1.0 (Winsteps® Rasch Measurement, 2017)

Figure 1 illustrated the plot of item loadings on the first factor extracted from the residuals, which separated the items into three clusters (1, 2, 3…). The plot graphically maps the loadings of items in the off-target dimension with item loadings on the two ends of the plot. The loadings of item 2(0.69), item 3(0.66), and item 1 (0.15) appeared to be another dimension, with item 5 (-0.69), item 7 (-0.56), and item 4 (-0.10). However, the contrast of the item loadings in the passive function scale was not as strong (< 2), and the second dimension is unlikely. Moreover, the disattenuated correlation between clusters in passive items approached 1.000, indicating that the two clusters of items were measuring the same thing.

The person reliability was acceptable (0.70), while Cronbach’s alpha was 0.83. The item reliability was excellent (0.97). No disordered category was found, however, a disordered threshold was found for item 1 (Cleaning palm), item 2 (Cutting fingernails), item 3 (Putting on a glove), item 6 (Put on a splint), and item 7 (Positioning arm on a cushion or support in sitting) (Table 2). The ArmA-TH passive function seemed not to be well-targeted in this sample, as the mean logit between item and person was more than 1 SD. Item bias or DIF was not found for ArmA-TH passive function.

**Table 2:** Rasch analysis results of ArmA-TH passive and active function sub-scale

**Section A**^a^ **Passive function sub-scale**	**Measure**	**Infit**	**Outfit**	**Disordered threshold**	**Local dependence**
1. Cleaning palm	0.08	0.93	0.89	Yes	No
2. Cutting fingernails	-0.79	0.79	0.77	Yes	No
3. Putting on a glove	-0.49	0.96	0.86	Yes	No
4. Cleaning armpit	-0.03	0.8	0.73	No	No
5. Putting arm through a sleeve	0.07	1.21	1.23	No	No
6. Put on a splint	1.06	1.31	0.89	Yes	No
7. Positioning arm on a cushion or support in sitting	0.09	1.19	1.31	Yes	No
**Section B**^b^ **Active function sub-scale**					
1. Fasten buttons on clothing	-0.39	0.96	0.87	Yes	No
2. Pick up a glass, bottle, or can	1.15	0.86	1.11	Yes	No
3. Use a key to unlock the door	-0.06	0.91	0.8	Yes	No
4. Write on paper	-0.54	1.22	1.22	Yes	No
5. Open a previously opened jar	-0.39	0.92	0.92	Yes	No
6. Eat with a knife and fork	0.55	0.94	0.8	Yes	No
7. Hold an object still while using unaffected hand	-1.66	1.38	1.75	Yes	No
8. Difficulty with balance when walking due to your arm	-0.87	1.12	1.27	Yes	No
9. Dial a number on home phone	-0.2	1.07	0.88	Yes	No
10. Tuck in your shirt	-0.36	1.19	1.3	Yes	No
11. Comb or brush your hair	0.92	0.64	0.35	Yes	#12 #13
12. Brush your teeth	0.89	0.68	0.57	Yes	#13
13. Drink from a cup or mug	0.95	0.72	0.41	Yes	#12

^a^Asks about ‘caring’ for your affected arm either yourself with your unaffected arm or by a carer or a combination of both of these. This section does not ask about using your affected arm to complete any of the tasks. ^b^Asks what you can do with your affected arm or using both arms

Based on the disordered threshold of each item (not shown here), it was suggested that categories 0–1 and 3–4 be collapsed, which reduced the responses from 5 to 3.

### Reanalysis after rescoring from 5 to 3 response options; 0 + 1, 2, 3 + 4

The eigenvalue of the first construct was reduced to 1.52 (12.6 %). The infit MNSQ or outfit MNSQ ranged from 0.82 (item 2) to 1.41 (item 5) (Table 3). The disattenuated correlation between person measures was 1.00, and no local dependence was found, all of which suggested unidimensionality. The person reliability was reduced to 0.51. The Cronbach’s alpha was 0.82. The item reliability was excellent (0.96). No disordered category threshold was found after this reanalysis.

**Table 3:** Rasch analysis results of ArmA-TH passive and active function sub-scale after rescoring

**Section A**^a^ **Passive function sub-scale**	**Measure**	**Infit**	**Outfit**
1. Cleaning palm	0.28	0.98	0.94
2. Cutting fingernails	-1.59	0.87	0.89
3. Putting on a glove	-1.11	0.84	0.79
4. Cleaning armpit	0.02	0.90	0.84
5. Putting arm through a sleeve	0.42	1.21	1.27
6. Put on a splint	1.68	1.11	0.87
7. Positioning arm on a cushion or support in sitting	0.30	1.20	1.37
**Section B**^b^ **Active function sub-scale**			
1. Fasten buttons on clothing	-0.35	0.84	0.92
2. Pick up a glass, bottle, or can	1.18	1.58	0.92
3. Use a key to unlock the door	-0.01	0.71	0.91
4. Write on paper	-0.58	1.18	1.25
5. Open a previously opened jar	-0.38	0.76	0.88
6. Eat with a knife and fork	0.30	0.95	1.11
7. Hold an object still while using unaffected hand	-1.61	1.50	1.15
8. Difficulty with balance when walking due to your arm	-0.79	1.67	1.05
9. Dial a number on home phone	-0.15	0.78	1.01
10. Tuck in your shirt	-0.42	0.96	1.04
11. Comb or brush your hair	0.96	0.32	0.67
12. Brush your teeth	0.93	1.30	0.75
13. Drink from a cup or mug	0.92	0.53	0.80

^a^Asks about ‘caring’ for your affected arm either yourself with your unaffected arm or by a carer or a combination of both of these. This section does not ask about using your affected arm to complete any of the tasks. ^b^Asks what you can do with your affected arm or using both arms.

### ArmA-TH active function

For the ArmA-TH active function, all items except item 7 fell within an acceptable range of fit indices. This implied that item 7 could derail the Rasch measurement model (Table 2). The principal component analysis of residuals showed the first construct with an eigenvalue of 2.57 (8.9 %), suggesting a violation of unidimensionality. The standardized residual correlations between items 13 and 12 was 0.59; between items 11 and 12 was 0.52; and between item 11 and item 13 was 0.45, indicating local dependence, as depicted, and could be a source for another dimension (Table 2).

**Fig. 2:**
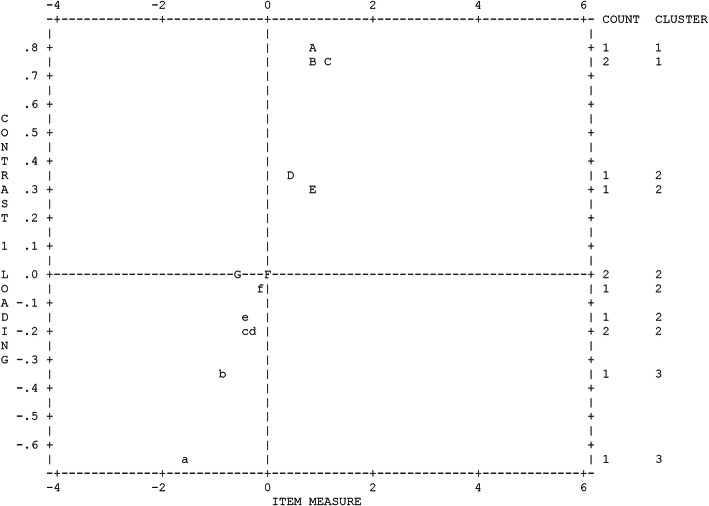
Plots of item loadings on the first factor of active function scale. Legend: Alphabet A–G and a–f represent 17 items on the active function scale. Figure created by Winsteps 4.7.1.0 (Winsteps® Rasch Measurement, 2017)

Figure 2 illustrates the plot of item loadings on the first factor extracted from the residuals, which separated the items into three clusters. The plot graphically maps the loadings of items in the off-target dimension with items with loadings at the two ends of the plot. Each letter (A, B, C,…a, b, c….). represents an item of the scale. The positive loadings are at the top end. A (0.83), B (0.81), and C (0.61), representing items 11, 12, and 13 appeared to be another dimension, with a (-0.51), b (-0.43), and c (-0.23) representing items 7, 5, and 3 with a strength of around 3 out of 13 items. However, the disattenuated correlation approached 1.000, therefore the person measures from the two clusters of items were statistically the same, indicating that the two clusters of items were measuring the same thing. To put it another way, the secondary dimension underlying the first contrast did not exist but was a strand.

The person reliability was acceptable (0.77), while Cronbach’s alpha was 0.85 and the item reliability was excellent (0.99). A disordered category was found in item 10 and disordered thresholds were found in all items. The ArmA-TH active function sub-scale did not appear to be well-targeted in this sample, as the mean logit between item and person was more than 1 SD. Item bias or DIF was not found in ArmA-TH active function for age or sex, or for different education levels.

### Reanalysis after rescoring from 5 to 3 response options; 0, 1 + 2, 3 + 4

The eigenvalue of the first construct was reduced to 2.23 (8.5 %). The infit MNSQ or outfit MNSQ ranged from 0.32 (item 11) to 1.67 (item 8) (Table 3). Notably, the outfit MNSQ of item 7 was reduced, whereas that of item 8 increased. The standardized residual correlations between items 13 and 12 was 0.28; between items 11 and 12 was 0.33, and between items 11 and item 13 was 0.61, indicating some local dependence. However, the disattenuated correlation between person measures was 1.00, suggesting sufficient unidimensionality. The person reliability increased to 0.71, Cronbach’s alpha was 0.84, and the item reliability was excellent (0.98).

Transformation of raw scores to Rasch-scaled scores using the original 5 responses is illustrated in Table 4. Ideally, the ArmA raw score should be converted to the Rasch-scale score on the users’ own data. However, this converted logit-scale should be applicable to situations where the data exhibit a similar fit to the model.

**Table 4:** Transformation of raw ArmA-TH scores to logits and then rescored to the original scale (*n* = 185)

Raw score	scale score	Raw score	scale score	Raw score	scale score
**ArmA-TH: Passive function**					
0	0	10	12	20	17
1	4	11	13	21	17
2	6	12	13	22	18
3	8	13	14	23	19
4	9	14	14	24	19
5	9	15	14	25	20
6	10	16	15	26	22
7	11	17	15	27	24
8	11	18	16	28	28
9	12	19	16		
**ArmA-TH: Active function**					
0	0	18	22	36	28
1	7	19	22	37	28
2	10	20	23	38	29
3	12	21	23	39	29
4	14	22	23	40	30
5	15	23	24	41	30
6	16	24	24	42	31
7	16	25	24	43	32
8	17	26	24	44	32
9	18	27	25	45	33
10	18	28	25	46	34
11	19	29	25	47	35
12	19	30	26	48	37
13	20	31	26	49	39
14	20	32	27	50	41
15	21	33	27	51	45
16	21	34	27	52	52
17	21	35	28		

## Discussion

This study aimed to explore the measurement and scaling properties of the ArmA-TH using Rasch analysis in patients with hemiparetic upper limbs. Our findings confirmed the unidimensonality of both the passive and the active function sub-scales. We found the same items to have a disordered threshold, as did Ashford et al. (except for item 2). Although rescoring seemed to make the data fit the Rasch measurement model, this risks reducing person reliability in the passive sub-scale, which has fewer items compared with the active sub-scale. However, some investigators have been less concerned by the disordered threshold because it does not impact construct validity [8].

For the active function sub-scale, we found the original data did not fit well with the Rasch measurement model when compared with the passive function sub-scale. We assume that the poor fit comes from two sources. First, some items do not contribute sufficiently to the construct. Four items were identified to be problematic. Item 7 ‘Hold an object still while using unaffected hand’ did not contribute to the same construct as the other items. The high value of misfit indicated that this item was not productive, albeit not harmful to the overall scale. Although items 11, 12, and 13 were dependent on each other to the extent that they could form a second dimension, the disattenuated correlation between person measures on the two item clusters suggested that they measured the same thing.

Second, all items in the active function sub-scale were found to have disordered thresholds; rescoring from 5 to 3 response options improved the fit with the Rasch model and was acceptable. The possible reason for the disordered thresholds might relate to a limited comprehension of the rating scale by stroke patients due to cognitive impairment, which is found in 20–80 % of post stroke patients and is present as early as 3–6 months after stroke onset [15, 16]. The previous study showed that the active function scale fitted in non-parametric item response theory (Mokken analysis), but not with the present study using a stricter model as a Rasch model [2]. This means that the possibility of using a sum score to produce a reasonable person measure on an interval scale may not be completely accurate. Using a Rasch model creates an opportunity to identify some potential problematic items.

Further exploration of category and threshold adjustment should be carefully considered, particularly for the passive function sub-scale, which had fewer items, rendering a low level of person reliability. While related studies have shown that a disordered threshold does not cause much problem for the model compared with some misfitting items [17, 18], we found that rescoring seemed not to improve the fit to the model. Therefore, we preferred keeping the original 5-response options.

One limitation of the study was that the larger sample size still needed for further analysis to ascertain the fitting of the data to the Rasch model, e.g. 400, as recommended by experts [19].

Another limitation was that some participants required assistance to read the questionnaires because of visual or physical impairment. The assistance might have influenced their responses or interfered with their freedom to respond.

## Conclusions

According to results of the Rasch analysis, both ArmA-TH active and passive function sub-scales data fit the Rasch model. Even though item 7 of the active function sub-scale seemed to present extra challenge for the Rasch model, the item was not considered harmful to the overall measurement, provided useful clinical information, and was therefore retained. It is worth noting that a better fit to the model was observed when the item responses were rescored from 5 to 3. Rescoring the item responses to less than 5 should be considered in future evaluations of ArmA-TH. Poor targeting in this sample implied that more easier items assessing arm function should be added [2, 19].

## Data Availability

The datasets used and/or analysed during the current study are available from the corresponding author on reasonable request.

## Abbreviations

ArmA: Arm Activity Measure
ArmA-TH: Arm Activity Measure Thai version
CIT: Classic Test Theory
DIF: Differential Item Functioning
IRT: Item Response Theory
I-CVI: Content Validity Index for Item
S-CVI: Content Validity Index for Score

## References

[1] Ashford S, Slade M, Turner-Stokes L (2013). Conceptualisation and development of the arm activity measure (ArmA) for assessment of activity in the hemiparetic arm. Disabil Rehabil.

[2] Ashford S, Turner-Stokes L, Siegert R, Slade M (2013). Initial psychometric evaluation of the Arm Activity Measure (ArmA): a measure of activity in the hemiparetic arm. Clin Rehabil.

[3] Buntragulpoontawee M, Euawongyarti P, Wongpakaran T, Ashford S, Rattanamanee S, Khunachiva J (2018). Preliminary evaluation of the reliability, validity and feasibility of the arm activity measure - Thai version (ArmA-TH) in cerebrovascular patients with upper limb hemiplegia. Health Qual Life Outcomes.

[4] Trevor G, Bond CM, Fox (2015). Applying the Rasch model: fundamental measurement in the human sciences.

[5] Ashford S, Siegert RJ, Alexandrescu R (2016). Rasch measurement: the Arm Activity measure (ArmA) passive function sub-scale. Disabil Rehabil.

[6] Linacre JM (2017). Winsteps® Rasch measurement computer program: user’s guide.

[7] Andrich D (2004). Controversy and the Rasch model: a characteristic of incompatible paradigms?. Med Care.

[8] Tennant A, Conaghan PG (2007). The Rasch measurement model in rheumatology: what is it and why use it? When should it be applied, and what should one look for in a Rasch paper?. Arthritis Rheum.

[9] Myers ND, Wolfe EW, Feltz DL, Penfield RD (2006). Identifying differential item functioning of rating scale items with the Rasch model: An introduction and an application. Meas Phys Educ Exerc Sci.

[10] Institute for Objective Measurement, Inc. Validity and Rasch Measurement: Construct, Content, etc. (2005). Accessed 30 Apr 2020.

[11] Christensen KB, Makransky G, Horton M (2017). Critical values for Yen’s Q3: Identification of local dependence in the Rasch model using residual correlations. Appl Psychol Meas.

[12] Andrich D (2013). An expanded derivation of the threshold structure of the polytomous Raschmodel that dispels any “Threshold disorder controversy”. Educ Psychol Meas..

[13] Gothwal VK, Wright TA, Lamoureux EL, Pesudovs K (2009). Rasch analysis of visual function and quality of life questionnaires. Optom Vis Sci.

[14] Smith EV (2002). Detecting and evaluating the impact of multidimensionality using item fit statistics and principal component analysis of residuals. J Appl Meas.

[15] Madureira S, Guerreiro M, Ferro JM (2001). Dementia and cognitive impairment three months after stroke. Eur J Neurol.

[16] Sun J-H, Tan L, Yu J-T (2014). Post-stroke cognitive impairment: epidemiology, mechanisms and management. Ann Transl Med.

[17] Adams RJ, Wu ML, Wilson M (2012). The Rasch rating model and the disordered threshold controversy. Educ Psychol Measur.

[18] Baghaei P, Hohensinn C, Kubinger KD (2014). Persian adaptation of foreign language reading anxiety scale: a psychometric analysis. Psychol Rep..

[19] Linacre J. Sample size and item calibration [or Person measure] stability (1994). Accessed 30 Dec 2020.

